# Supplementary material for: Ultrasound-guided puncture reduces bleeding-associated complications, regardless of calcified plaque, after endovascular treatment of femoropopliteal lesions, especially using the antegrade procedure: A single-center study
Source: PLoS One. 2021 Mar 12;16(3):e0248416. doi: 10.1371/journal.pone.0248416 (PMC7954350; doi:10.1371/journal.pone.0248416)
Supplement: Attachment — Submitted filename: Response to Reviewers.docx [file pone.0248416.s001.docx]

Reviewer#1

**1 why is this report that ultrasound guidance is important in vascular catheterization novel given references 15 and 16 and why is this of general conceptual importance?**

**a. is it calcification**

**b. is it antegrade**

**just not sure why these findings are so conceptually novel as to appeal to the broad readership of**

Thank you very much for your comment.

We apologize for the inadequate explanation.

In references 15 and 16, many risk factors for bleeding-associated complications have been verified, but the antegrade approach and puncture site calcification have not been verified. Although ultrasonic-guided puncture is greatly affected by artifacts in puncture site calcifications, it is novel in that it reduces the risk of bleeding-associated complications, especially in an antegrade puncture. In previous studies showing that ultrasound guide puncture is useful, many of the cases are of retrograde approaches. In modern EVT, the antegrade approach is often required, and the purpose was to retroactively confirm whether the ultrasound guide is effective in the recent EVT situation.

Therefore, we added the objective of this investigation in the Introduction section. (line 2-5, page 4)

**2 should it not be incidence of bleeding when US guidance is used and when not rather than was US guidance used in bleeding or not. Can the authors please provide that analysis? Otherwise we are dealing with small numbers**

Thank you very much for your comment.

The incidence of bleeding-associated complications with ultrasound-guided puncture was 0% (1/168), and the incidence without ultrasound-guided puncture was 14.4% (34/236). In this study, 236 (58%) of the total 404 cases did not undergo ultrasound-guided puncture. If ultrasound-guided puncture was performed in all cases, the incidence of complications is expected to decrease from the actual 35 cases to 2.4 cases. Therefore, we added the incidence of bleeding-associated complication in the Procedure section. (line 6-11, page 10)

**3 can you report relative risk not just odds ratio?**

I would like to thank you for the constructive comment.

The following data have been added to the revised manuscript. (line 2-6, page 12)

(Age ≥ 80 years): relative risk is 2.04

(Ultrasound-guided puncture): relative risk is 0.04

Reviewer#2

**1. The primary endpoint is not well defined - what is a "bleeding complication?" You say "main outcome measure of bleeding-associated complications was defined as bleeding at the**

**puncture site that required medical intervention" - what does this mean? What interventions were performed? This is poorly flushed out**

Thank you for your comment.

We apologize for the inadequate explanation.

The medical interventions required are compression, an extension of the bed rest period, transfusion, and operation for hemostasis. I added it in the Endpoints section. (line 3-4, page 5)

**2. How were these bleeding complications treated**

Thank you for your comment.

Of the 35 patients with bleeding-associated complications, all required manual re-compression and extension of the bed rest period, and 15 patients were treated with blood transfusions and treated without sequelae. No cases required operation for hemostasis.

Therefore, we added it in the Results section. (line 3-6, page 9)

**3. Overall tone of the text is very colloquial and should be revised**

Thank you very much for your comment.

I have revised the manuscript in accordance with your suggetion.
