# Supplementary material for: Ultrasound-guided puncture reduces bleeding-associated complications, regardless of calcified plaque, after endovascular treatment of femoropopliteal lesions, especially using the antegrade procedure: A single-center study
Source: PLoS One. 2021 Mar 12;16(3):e0248416. doi: 10.1371/journal.pone.0248416 (PMC7954350; doi:10.1371/journal.pone.0248416)
Supplement: Attachment — Submitted filename: Response_to_Reviewers.docx [file pone.0248416.s002.docx]

**Reviewer #1: The authors have answered my queries and yet I still remain unconvinced that there is much novel here or that there is a mechanistic, conceptual or scientific aspect to this study.**

Thank you very much for your comment. We have added the following information to the revised manuscript (page 12, line 12): “Several previous studies have demonstrated that ischemia of the ipsilateral lower extremity is associated with hemostasis after femoral arterypuncture [18, 21]. Therefore, antegrade cannulation, which is a puncture of the lower limb on the lesion side, requires safe puncture without complications. Most of previous studies have demonstrated the usefulness of ultrasound-guided puncture; however, the rates of antegrade cannulation were as low as 0–16% [15, 16, 22, 23], which were not sufficient to evaluate the usefulness of ultrasound-guided puncture in antegrade cannulation. In this study, 48% of patients had antegrade cannulation, which indicates that ultrasound guidance is useful even in patients with a relatively high number of ipsilateral antegrade cannulations.“

**Reviewer #2: All comments have been addressed. Ok to accept this paper. I have no further comments to add.**

Thank you very much for reviewing our manuscript.

**Reviewer #3: (No Response)**

Thank you very much for reviewing our manuscript.
